# α-Hispanolol Induces Apoptosis and Suppresses Migration and Invasion of Glioblastoma Cells Likely *via* Downregulation of MMP-2/9 Expression and p38MAPK Attenuation

**DOI:** 10.3389/fphar.2019.00935

**Published:** 2019-09-03

**Authors:** Vanesa Sánchez-Martín, Lidia Jiménez-García, Sandra Herranz, Alfonso Luque, Paloma Acebo, Ángel Amesty, Ana Estévez-Braun, Beatriz de las Heras, Sonsoles Hortelano

**Affiliations:** ^1^Unidad de Terapias Farmacológicas, Área de Genética Humana, Instituto de Investigación de Enfermedades Raras (IIER), Instituto de Salud Carlos III, Madrid, Spain; ^2^Departamento de Farmacología, Farmacognosia y Botánica, Facultad de Farmacia, Universidad Complutense de Madrid (UCM), Madrid, Spain; ^3^Departamento de Química Orgánica, Instituto Universitario de Bio-Orgánica Antonio González, Universidad de La Laguna, La Laguna, Tenerife, Spain

**Keywords:** α-hispanolol, apoptosis, glioblastoma, caspases, migration, matrix metalloproteinases

## Abstract

α-Hispanolol (α-H) is a labdane diterpenoid that has been shown to induce apoptosis in several human cancer cells. However, the effect of α-H in human glioblastoma cells has not been described. In the present work, we have investigated the effects of α-H on apoptosis, migration, and invasion of human glioblastoma cells with the aim of identifying the molecular targets underlying its mechanism of action. The results revealed that α-H showed significant cytotoxicity against human glioma cancer cell lines U87 and U373 in a concentration- and time-dependent manner. This effect was higher in U87 cells and linked to apoptosis, as revealed the increased percentage of sub-G_1_ population by cell cycle analysis and acquisition of typical features of apoptotic cell morphology. Apoptosis was also confirmed by significant presence of annexin V-positive cells and caspase activation. Pretreatment with caspase inhibitors diminishes the activities of caspase 8, 9, and 3 and maintains the percentage of viable glioblastoma cells, indicating that α-H induced cell apoptosis through both the extrinsic and the intrinsic pathways. Moreover, we also found that α-H downregulated the anti-apoptotic Bcl-2 and Bcl-xL proteins and activated the pro-apoptotic Bid and Bax proteins. On the other hand, α-H exhibited inhibitory effects on the migration and invasion of U87 cells in a concentration-dependent manner. Furthermore, additional experiments showed that α-H treatment reduced the enzymatic activities and protein levels of matrix metalloproteinase MMP-2 and MMP-9 and increased the expression of TIMP-1 inhibitor, probably *via* p38MAPK regulation. Finally, xenograft assays confirmed the anti-glioma efficacy of α-H. Taken together, these findings suggest that α-H may exert anti-tumoral effects *in vitro* and *in vivo* through the inhibition of cell proliferation and invasion as well as by the induction of apoptosis in human glioblastoma cells. This research describes α-H as a new drug that may improve the therapeutic efficacy against glioblastoma tumors.

## Introduction

Glioblastoma multiforme (GBM) is a rare disease with an incidence of 2–3 per 100,000 people. It is considered as one of the most malignant primary brain tumors in adults being associated with a bad prognosis. Despite standard treatments (surgery, radiotherapy, and chemotherapy), patient survival remains poor, as reflected by a median overall survival of less than 15 months (Stupp et al., 2005; Stupp et al., 2009). Resistance to apoptotic cell death, supported angiogenesis, and glioma cell invasion are believed to be the main mechanisms by which GBM shows resistance against conventional chemotherapy (Onishi et al., 2011). Nevertheless, the exact procedures driving GBM pathogenesis are not well known. Therefore, further research is required to understand the underlying mechanisms of tumor progression in order to design more effective therapies.

Natural products are one of the major sources of novel compounds for the treatment of many human diseases, including cancer. Indeed, an increasing interest to explore the anticancer effects of natural products in glioblastoma has emerged in last years (Park et al., 2017; Zhao et al., 2017; Vengoji et al., 2018). Hispanolone is a labdane diterpenoid that was first isolated from *Ballota hispanica*, a Labiatae species growing in Spain (Savona et al., 1978). Hispanolone derivatives have been reported to exhibit anti-inflammatory and anti-tumoral effects (Giron et al., 2008; Traves et al., 2013; Mota et al., 2015). Thus, they have been shown to inhibit the growth and to induce apoptosis in various types of human cancers (Traves et al., 2013; Mota et al., 2015). Hispanolone derivatives, such as α-H, induce cell death of several tumor cells including melanoma, leukemia, hepatocellular carcinoma, and breast by activating caspase signaling and modifying the levels of pro-apoptotic and anti-apoptotic factors (Traves et al., 2013). Furthermore, these compounds also sensitize hepatocellular carcinoma cells to TRAIL-mediated apoptosis through upregulation of the death receptors DR4 and DR5 (Mota et al., 2015). Interestingly, hispanolone derivatives also inhibited tumor growth of melanoma and ovarian cells *in vivo* (Traves et al., 2013). Nevertheless, the anti-tumoral effects of α-H on glioblastoma cells remain unclear. Therefore, the aim of this study was to investigate the efficacy of α-H against glioma progression using *in vitro* and *in vivo* models. We showed that α-H increased apoptosis and reduced invasion and migration of glioma cells. In addition, we demonstrated that activities of MMP-2 and MMP-9 were significantly inhibited by α-H treatment, whereas TIMP-1 expression was increased. Further studies revealed that MMP expression might be regulated by the protein kinase p38MAPK. Finally, we also found that α-H inhibited *in vivo* tumor growth in mice subcutaneous xenograft, which was linked to impaired p38MAPK phosphorylation and reduced MMPs expression. Taken together, our data provide evidence that α-H may be a useful therapeutic agent for GBM treatment.

## Materials and Methods

### Reagents

Western blot reagents were obtained from GE Healthcare (Pittsburgh, PA, USA). Fluorescent probes for caspase activity, caspase inhibitors, and annexin V assay kit were from BD Biosciences (San José, CA, USA). Culture media were from Lonza (Basel, Switzerland). MTT (3-[4,5-dimethylthiazol-2-yl]-2,5-diphenyl tetrazolium bromide) and p38MAPK inhibitor (SB202190) were obtained from Sigma-Aldrich (St. Louis, MO, USA). Primary monoclonal rabbit antibodies against caspase 8 (dilution, 1:1,000; #4927), cleaved-caspase 9 (dilution, 1:1,000; #7237), MMP-2 (dilution, 1:1,000; #4022), MMP-9 (dilution, 1:1,000; #3852), p-p38 (dilution, 1:1,000; #9211), p38 (dilution, 1:1,000; #9212), and TIMP-1 (dilution, 1:1,000; #8946) were purchased from Cell Signaling Technology, Inc. (Danvers, MA, USA). Caspase 3 (dilution, 1:1,000; sc-7148), Bid (dilution, 1:1,000; sc-11423), Bcl-2 (dilution, 1:1,000; sc-783), Bcl-xL (dilution, 1:1,000; sc-634), and Bax (dilution, 1:1,000; sc-526) were purchased from Santa Cruz Biotechnology, Inc. (Dallas, TX, USA), and β-actin (dilution, 1:5,000; #A5441) was obtained from Sigma-Aldrich (St. Louis, MO, USA).

Anti-Ki67 (dilution 1:200) and Click-iT Tunel colorimetric IHC detection kit were from Thermo Fisher (Waltham, MA USA). DAB kit was provided from Vector laboratories (Burlingame, CA, USA).

### Preparation of α-Hispanolol

α-hispanolol (α-H) was obtained from the natural diterpene hispanolone as previously reported (Giron et al., 2008) following the procedure described by Rodríguez-Hahn et al. (1995) (**Supplementary Data 1** and **Figure S1**). The purity of α-H is higher than 99%. The corresponding ^1^H-NMR and ^13^C-NMR data are shown (**Supplementary Figures S2, S3**).

Stocks of α-H were prepared in DMSO and diluted in PBS before use (vehicle, maximum DMSO concentration 0.01%).

### Cell Lines

Human glioma cell lines U87 and U373 and microglial BV2 cell line were cultured in DMEM supplemented with fetal bovine serum (10% FBS) and 100 U/ml penicillin and 100 μg/ml streptomycin. Cell lines were tested for mycoplasma using a Mycoplasma Detection Kit (Lonza) and stored in liquid nitrogen until use.

### MTT Assay for Cell Viability

Cells were incubated in triplicate in the presence of different concentrations of α-H for 24, 48, or 72 h. After treatment, 10 μl of MTT solution (5 mg/ml) was added to each well, and the cells were then incubated at 37°C for 3 h. The insoluble product, formazan, was solubilized using DMSO, and the absorbance was read at 570 nm. Results are expressed as the percent reduction in cell viability compared with untreated control cultures for at least three independent experiments. The IC_50_ values were calculated by GraphPad Prism5.0 software.

### Colony Formation Assays

U87 cells were seeded in six-well plates (5 × 10^2^ cells/well) and allowed to adhere for 24 h before treatment. Then, cells were treated with α-H (10 μM) or vehicle as control for 48 h and were cultured for 14 additional days changing culture medium with α-H every 3 days. Then, cells were washed with PBS, fixed with 3% formaldehyde for 10 min, and permeabilized with 2% methanol for 2 min. Colonies were stained with 0.5% crystal violet in 20% methanol for 1.5 min, washed with running water to remove excess dye, and let wells air-dried. Relative clonogenicity of the diterpenoid-treated cells was computed as percentage of the number of colonies that formed compared with vehicle treated wells.

### Cell Cycle Analysis

Cell cycle analysis was assessed by flow cytometry after staining with propidium iodide. After treatment of U87 cells with different concentrations of α-H, cells were washed twice in PBS and fixed in 70% ethanol. DNA was stained with 50 μg/ml propidium iodide (Sigma-Aldrich) and RNase A (1 mg/ml, Sigma-Aldrich) for 30 min at 37ºC in a dark environment, and fluorescence was measured using a MACSQuant (Miltenyi Biotics; Bergisch Gladbach, Germany).

### Annexin-V Binding Assay for Apoptosis

α-H-induced cell apoptosis was assessed using an annexin V apoptosis detection kit according to manufacturer’s protocol (BD Pharmingen). Briefly, cells (2 × 10^5^), both adherent and floating, were harvested after treatment with different concentration of α-H, washed twice with PBS, and resuspended in 100 μl of binding buffer. Annexin V-FITC was added to individual samples and incubated for 15 min under darkness. Then, 400 μl of 1X binding buffer was added to stop the reaction, and after adding propidium iodide, samples were analyzed by flow cytometry with MACSQuant (Miltenyi Biotec).

### Cell Morphology

Cells were grown to 70% confluence and treated with different concentrations of α-H for 24 h. Changes in cell morphology were imaged using an inverted phase contrast microscope. Representative images from each case are shown.

### Total Extracts and Western Blot Analysis

Cell lysates were prepared at 4°C using ice-cold buffer of total extract (0.5% Chaps, 10mM Tris pH 7.5, 1 mM Cl_2_Mg, 1 mM EGTA, 10% glycerol, 5 mM β-mercaptoethanol, and 1 μl/ml of proteinase cocktail inhibitor), scraping off the plate and maintaining for 15 min under continuous shaking. After centrifugation, supernatants were collected, and solubilized proteins were quantified using a BCA protein assay kit (Pierce). Protein extracts were electrophoresed on SDS-PAGE (10–15% gels) and transferred onto polyvinylidenedifluoride membranes (GE Healthcare), which were incubated with the following antibodies: caspase 8, caspase 3, cleaved caspase 9, Bid, Bcl-2, Bcl-xL, Bax, MMP-2, MMP-9, TIMP-1, p-p38MAPK, p38MAPK, and β-actin. Membranes were incubated with HRP-conjugated secondary antibody. Enzymatic signal was detected using an enhanced chemiluminescence kit (Immobilon Westerns, Millipore, Merck, Darmstadt, Germany). β-actin was used as a loading control. Relative protein expression was analyzed by densitometry using Quantity One software (Bio-Rad, CA, USA). For all experiments, representative Western blots displaying results obtained from three independent experiments are shown.

### Caspase Assays

The activity of caspase 3, 8, and 9 was determined in protein extracts using the fluorimetric substrates Ac-DEVD-AMC, Ac-IETD-AFC, and Ac-LEHD-AMC, respectively, according to the manufacturer’s instructions (BD Biosciences, San José, CA, USA).

### Wound-Healing Assay

U87 cells were cultured in 24-well plates and grown to sub-confluence. Then, cell monolayers were carefully scratched using a sterile pipette tip to create a linear “wound” in each well. Plates were washed twice with PBS to remove cell debris and incubated with indicated concentrations of α-H or vehicle as control. Wound healing process was monitored by photomicroscopy at different time points and quantified with Image J software.

### Transwell Migration Assay

Cell invasion was determined using a transwell system (Boyden chamber, 8-μm pore size) from BD Biosciences (San Diego, CA, USA). Twenty thousand cells were placed on upper wells in serum-free medium in the presence or absence of amounts of α-H described in the figure. Migration-inducing medium containing 10% FBS was added to lower wells. After 48 h in a cell culture incubator, filters were washed twice with PBS, fixed, and crystal violet stained as described previously. Next, cells that did not migrate and were found on the upper side of the membrane were discarded by wiping with a cotton swab. Transmigrated cells to the lower side of the membrane were evaluated by: 1) light field microscopy through scanning randomly four fields per filter. 2) Spectrophotometry after dissolving crystal violet with 0.1 M sodium citrate, 50% ethanol, pH 4.2, and measure absorbance at 540 nm, which represent percentage of migrated cells.

### Gelatin Zymography

MMP-2 and MMP-9 activities were determined by gelatin zymography. Briefly, cells were treated with vehicle or different concentrations of α-H for 24 h. Supernatants were collected to prepare samples with loading buffer under nonreducing denaturing conditions and separated on an 8% SDS-PAGE gel containing 0.1% gelatin. After electrophoresis, gels were washed twice in 100 ml of 2% Triton X-100 to remove SDS and incubated in activation buffer (50 mM Tris–HCl, pH 7.8, 200 mM NaCl, and 5 mM CaCl_2_) at 37°C overnight. Then, gel was stained with 0.5% Coomassie Brilliant Blue R250 for 1 h and destained with washing buffer (10% methanol and 5% glacial acetic acid) until clear bands suggestive of gelatin digestion were detected against the blue background. The gelatinolytic activities were quantified and analyzed by Quantity One software (Biorad, CA, USA).

### Real-Time Quantitative RT-PCR

U87 cells were treated with vehicle or α-H for 24 h, and total RNA extraction was performed using the Trizol reagent (Life Technologies, Carlsbad, CA) following manufacturer’s recommendations. RNA concentration was quantified with an ultraviolet spectrophotometer (Nanodrop). cDNA was synthesized from 1 μg of total RNA using SuperScript™ III First-Strand Synthesis SuperMix (Life Technologies, Carlsbad, CA) according to the manufacturer’s instructions. Specific primers used for the PCR reaction are as follows: MMP-2, 5′-TGATGGTGTCTGCTGGAAAG-3’ (forward), 5′-CTACAGGACAGAGGGACTAGAG-3′ (reverse); MMP-9, 5′-CATTCAGGGAGACGCCCATT-3′ (forward), 5′-AACCGAGTTGGAACCACGAC-3′ (reverse); GAPDH, 5′- TCACTGCCACCCAGAAGA-3′ (forward), 5′-TACCAGGAAATGAGCTTGAC-3′ (reverse), TIMP-1, 5´-AGAGTGTCTGCGGATACTTCC-3´ (forward), 5´-CCAACAGTGTAGGTCTTGGTG-3´ (reverse). Quantitative PCR (SYBR Green) analysis was performed on an ABI 7500 Fast sequence analyzer (Applied Biosystems, Darmstadt, Germany). Each sample was run in duplicate, and the relative mRNA levels of MMP-2 and MMP-9 were normalized to GAPDH as internal control. The experiment was performed for three times.

### Generation of Tumor Xenografts

All animal care and experimental procedures were conducted in accordance with the guidelines for Animal Care and were approved by the Animal Committee of the Instituto de Salud Carlos III. Studies involving animals are reported in accordance with the ARRIVE guidelines for reporting experiments involving animals (Kilkenny et al., 2010; McGrath et al., 2010). For heterotopic/subcutaneous xenografts, 3 × 10^6^ U87 cells resuspended in 100 µl of PBS were subcutaneously injected in the right flank of 5-week-old immunodeficient NSG (NOD scid gamma) mice. Once tumors reached approximately an average size of 200 mm^3^, the animals were randomly divided into the following experimental groups: those that were intravenous injected with 1 mg/kg body weight of α-H or those that received vehicle control on day 12, 15, and 18. Animals were monitored daily, and body weight was taken. Tumor sizes were measured once every 2 days using a calliper, and volume was calculated as tumor size = 1/2(*length* × *width*^2^). After 21 days, the mice were sacrificed, and the tumors were excised and stored at -80°C until further analysis.

### Immunohistochemical Analysis of Tumors

Tumor tissues were harvested and embedded in paraffin, and 5-μm cryostat sections were obtained. Expression of Ki67 was detected after incubation with anti-Ki67 antibody (1:200; Thermo Fisher) and visualized using a colorimetric method (DAB kit; Vector Laboratories). Apoptotic cells were detected in tissues using Click-iT Tunel colorimetric IHC detection kit (Invitrogen). Five randomly selected anonymous tumor fields of each specimen were evaluated for positive cells by three independent investigators.

### Statistical Analysis

All data are presented as mean ± standard deviation (S.D.) of at least three independent experiments. Data were analyzed using GraphPad Prism 5.0 (GraphPad Software, CA, USA). Statistical significance was estimated by Student’s *t* test for comparison between two groups. For comparison between two or more groups, one-way ANOVA, followed by Turkey’s multiple comparisons test was used. Tumor volumes of the treated groups were compared with those of the control group using a paired *t* test. Values at *p* < 0.05 were considered as statistically significant.

## Results

### α-H Decreased Cell Viability and Colony Formation in Glioblastoma Cells

**Figure 1:**
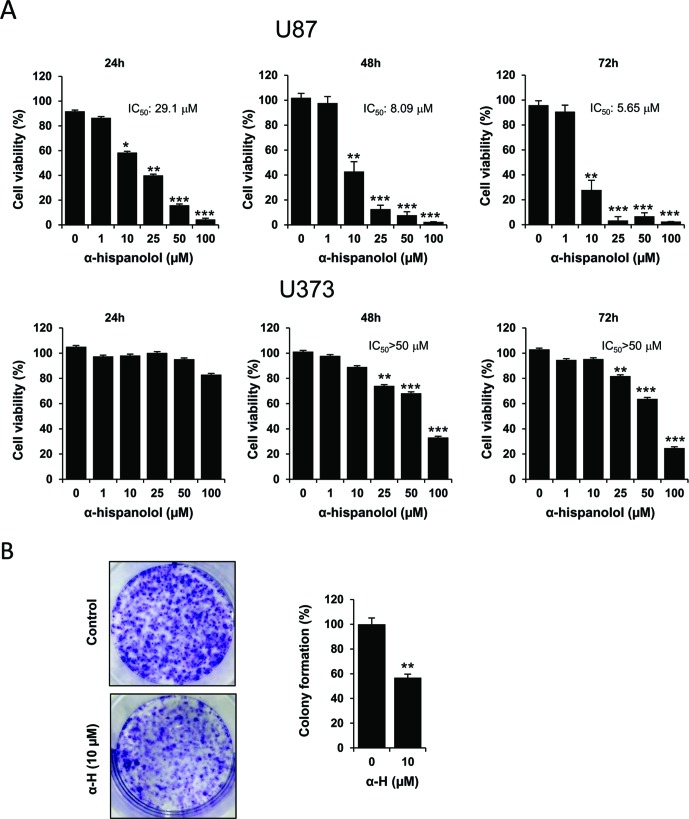
α-H inhibited proliferation and suppressed colony formation in glioblastoma cells. **(A)** U87 and U373 cells were treated with different concentrations of α-H (1–100 μM) for 24, 48, and 72 h. Cell viability was determined by MTT assay and reported as mean of the cell viability percentage ± S.D. from at least three independent experiments. *P < 0.05, **P < 0.01, and ***P < 0.001 with respect to control. **(B)** Colony formation of U87 cells treated with α-H (10 μM) was evaluated by clonogenic assay. Representative images of crystal violet-stained cultures are shown on the left. Bar graph shows mean of the colony formation percentage ± S.D. from three independent experiments. **P < 0.01 with respect to vehicle-treated cells.

To explore the cytotoxic effects of α-H on glioblastoma cells, U87 and U373 lines were treated for 24, 48, and 72 h with 1, 10, 25, 50, and 100 μM of α-H, and cell viability was determined by MTT assay (**Figure 1A**). The viability of cells treated with α-H decreased in a dose-dependent manner. In U87 cells, half maximal inhibitory concentration (IC_50_) of α-H was 29.1 µM after 24 h, which declined to 5.65 µM at 72 h. In comparison with U87 cells, U373 cells were less sensitive to α-H in all tested conditions, even showing an IC_50_ value higher than 50 μM at 72 h. These results indicate that α-H exhibited more prominent effects on U87 cells, and we focused on these cells for further experiments. The cytotoxic effect of α-H was further confirmed using a clonogenic assay. As shown in **Figure 1B**, treatment with α-H also inhibited the colony forming ability of these cells.

### Effects of α-H on Cell Cycle and Apoptosis in U87 Cells

**Figure 2:**
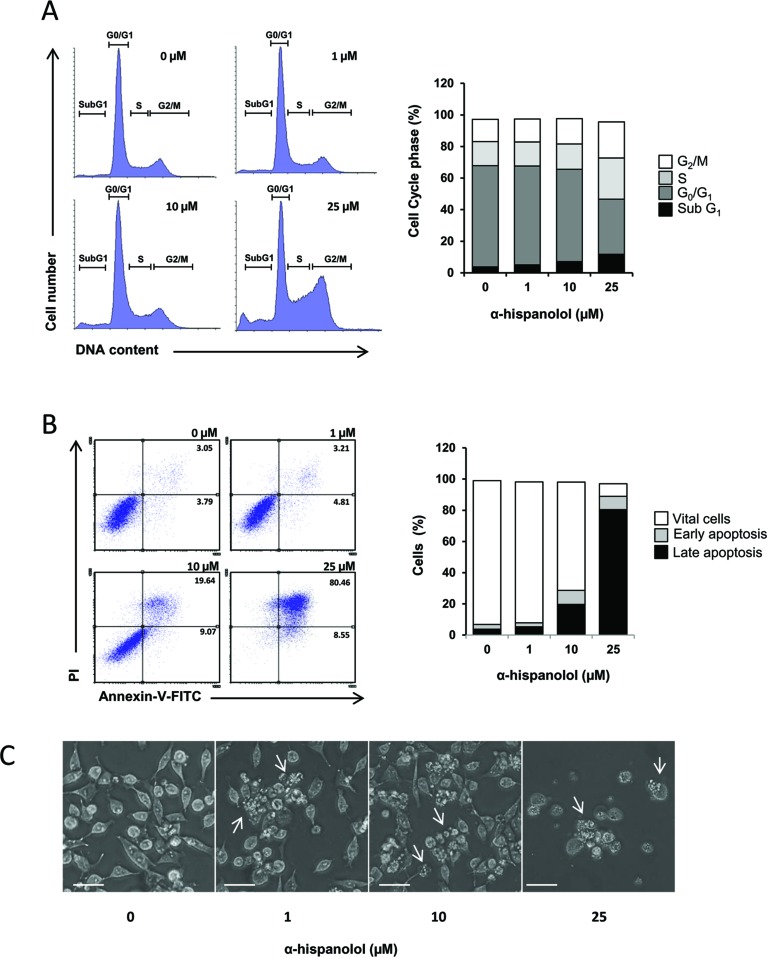
Effects of α-H on cell cycle and apoptosis in U87 cells. **(A)** U87 cells were treated with different concentrations of α-H (1, 10, and 25 μM) or vehicle as control for 24 h, then fixed in ethanol, and stained with propidium iodide. DNA content was determined by flow cytometry. Percentage of cells in each phase of the cell cycle (sub-G_1_, G_0_/G_1_, S, and G_2_/M) showed in the bar graph were calculated using Flowing software. **(B)** U87 cells were treated with different concentrations of α-H (1, 10, and 25 μM) or vehicle as control for 24 h. Collected cells were stained with annexin V-FITC and propidium iodide, and then analyzed by flow cytometry. Numbers in the plots represent percentage of the correspondent apoptotic population. Graph bars show percentage of correspondent cellular phenotype. **(C)** Microphotographs were taken after 24 h of incubation with different concentrations of α-H or vehicle as control. Characteristics of apoptosis such as cell rounding (due to loss of adhesion to substratum) and plasma membrane blebbing are observed in α-H-treated cells (arrows) (scale bars = 40 µm). Data presented are from one representative experiment out of three.

To further explore the effect of α-H on cell proliferation and survival, flow cytometry measurements were performed to evaluate changes in cell cycle and apoptosis. Cells were treated with α-H (0, 1, 10, and 25 µM) and incubated for 24 h. Cell cycle analysis showed that α-H treatment significantly decrease the percentage of cells at G_0_/G_1_ phase followed by augmentation of the sub-G_1_ population, suggesting that α-H induced apoptosis (**Figure 2A**). To confirm that apoptosis is involved in the cell growth inhibition induced by α-H, U87 cells were treated with different concentrations of α-H, and apoptosis was determined by annexin V and PI binding assay. Results showed that percentage of apoptotic cells (early and late apoptosis) increased from 6.84% on vehicle-treated samples to 28.71% and 89.01% when incubated with 10- and 25-µM α-H, respectively (**Figure 2B**). Additionally, phase contrast microscopy revealed morphological changes of U87 cells after α-H treatment characterized by smaller, rounded, and some floated cells and the presence of plasma membrane blebbing (**Figure 2C**). According to the differences in sensitivity previously reported, treatment of U373 cells with α-H for 24 h did not show any significant changes in morphology or apoptosis (**Supplementary Figure S4**).

Furthermore, in order to confirm the specific effects of α-H on glioblastoma cells, we examined its toxicity on the non-tumoral microglial cell line BV2. Treatment of these cells with α-H at the same range of concentrations (1, 10, 25, 50, and 100 μM) did not show any significant effect on viability (as low as 14.5% reduction at 100 μM), exhibiting similar results when apoptosis was analyzed (**Supplementary Figure S5**).

### α-H Induced Glioblastoma Apoptosis *via* Caspase-Dependent Pathway and Regulation of Bax/Bcl-2 Ratio

**Figure 3:**
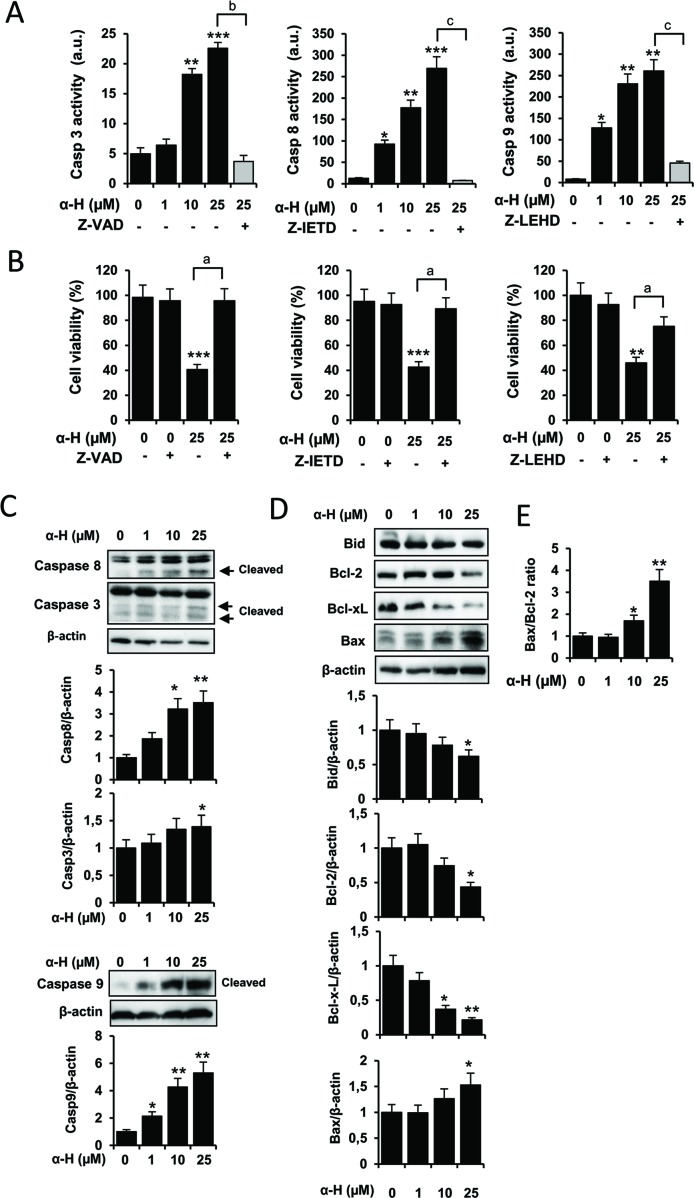
α-H induced apoptosis by regulating caspases and Bcl-2 family in human GBM cells. **(A)** U87 cells were treated for 24 h with different concentrations of α-H (1, 10, and 25 μM) or vehicle as control in the presence or absence of the indicated caspase inhibitors. Caspase 3, 8, and 9 activities were determined in cell extracts by fluorometry, using specific fluorigenic substrates as described in Materials and Methods. **(B)** U87 cells were treated for 24 h with 25-μM α-H or vehicle as control in the presence or absence of the caspase inhibitors indicated. Cell viability was determined by MTT assay. Results are reported as mean ± S.D. from three independent experiments. *P < 0.05, **P < 0.01, and ***P < 0.001 with respect to the non-treated cells. ^a^P < 0.05, ^b^P < 0.01, and ^c^P < 0.001 with respect to the α-H (25 μM) treated cells. **(C-D)** U87 cells were treated for 24 h with different concentrations of α-H (1, 10, and 25 μM) or vehicle as control, and caspases and members of Bcl-2 family protein levels were determined by Western blot. β-actin was used as loading control. A representative experiment of three performed is shown. Bars graphs show densitometry quantification of the bands from three independent experiments. **(E)** Bax/Bcl-2 ratio was calculated from data obtained by densitometry. *P < 0.05 and **P < 0.01 with respect to the non-treated cells.

Caspases are effector molecules of the apoptotic pathways classified by their mechanism of action as initiator (caspase 8 and 9) or executioner caspases (caspase 3). First, we examined activation of caspase 3 on α-H-induced apoptosis in U87 cells treated with different concentrations of the diterpenoid compound using the specific substrate Ac-DEVD-AMC. As shown in **Figure 3A**, caspase 3 activity was significantly induced by α-H in a dose-dependent manner. Co-incubation of cells with α-H (25 μM) in the presence of the pan caspase inhibitor Z-VAD-FMK suppressed the increased caspase 3 activity (**Figure 3A**). Interestingly, α-H treatment also induced activation of caspase 8 and 9 evaluated by using their specific substrates Ac-IETD-AFC and Ac-LEHD-AMC (**Figure 3A**). Additionally, cell death induced by α-H was also significantly reversed in the presence of Z-VAD and specific inhibitors for caspase 8 (Z-IETD-FMK) and caspase 9 (Z-LEHD-FMK) (**Figure 3B**). We further confirmed the effects of α-H on caspase activation by detecting their cleaved forms on Western blot analysis (**Figure 3C**).

Bid, a pro-apoptotic Bcl-2 family member, is cleaved by caspase 8, providing a link between death receptor and mitochondrial pathways of apoptosis (Billen et al., 2008). Furthermore, the prosurvival proteins Bcl-2 and Bcl-xL have been described to be upregulated in initial and recurrent glioblastoma patients, whereas downregulation of the pro-apoptotic protein Bax has been observed (Strik et al., 1999). In order to further explore potential signaling pathways by which α-H induced apoptosis, we evaluated the expression of the Bcl-2 family by Western blot (**Figure 3D**). Levels of Bcl-2 and Bcl-xL decreased after α-H treatment, whereas the pro-apoptotic Bax protein was upregulated, resulting in a significant increase in the ratio of Bax to Bcl-2 (**Figure 3E**). In addition, treatment with α-H diminished the inactive form of Bid.

### α-H Inhibited Migration and Invasion of U87 Cells

**Figure 4:**
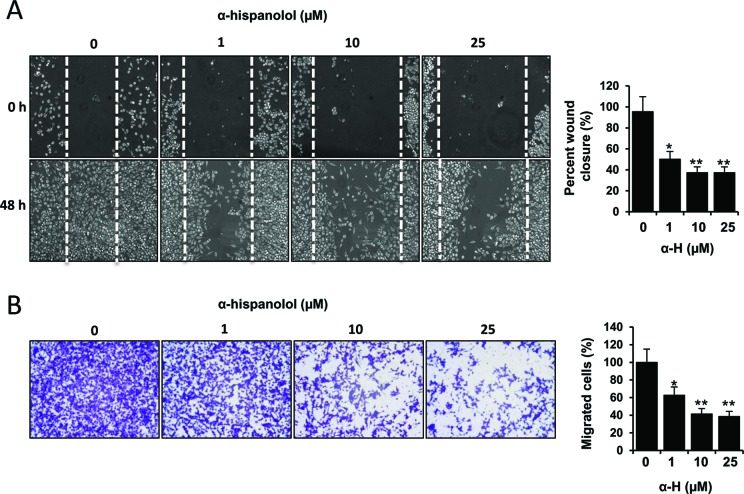
Effects of α-H on the motility and invasiveness of U87 cells. **(A)** Subconfluent U87 cultures were wound scratched and then incubated in media containing varying concentrations of α-H (1, 10, and 25 μM) or vehicle as control for 48 h. Cell migration was evaluated by photomicroscopy. Representative photographs showed the same area at time zero and after 48 h of incubation with or without α-H. Bars graph represents mean ± S.D. of percentage wound closure from three independent experiments. *P < 0.05 and **P < 0.01 with respect to the non-treated cells. **(B)** Cell invasion was determined using a transwell assay system. U87 cells treated with indicated concentrations of α-H (1, 10, and 25 μM) or vehicle as control were seeded into the upper chamber of the system in serum-free media. The bottom well was filled with complete medium as chemotactic attractant environment. After 48 h, cells migrated to the bottom side of the filter were fixed, crystal violet stained, and evaluated by light microscopy (left panel). Magnification, ×100. Invasion rate is quantified by spectrophotometry after eluting cell staining, as described in Materials and Methods. Values represent the mean percentage of migrated cells ± S.D. from three independent experiments performed in triplicate. *P < 0.05 and **P < 0.01 with respect to the non-treated cells.

One of the major causes of malignancy in GBM is the highly invasive ability of tumor cells (Tate and Aghi, 2009; Onishi et al., 2011). Accordingly, we next evaluated the role of α-H on migration and invasion of U87 cells by wound healing scratch and transwell invasion methods, respectively. U87 cells treated with α-H displayed reduced mobility into the wound area. Treatment with α-H significantly reduced cell migration in a concentration-dependent manner; the inhibition rates were ∼50.26%, 62.56%, and 63.74% with 1-, 10-, and 25-µM α-H, respectively (**Figure 4A**). These data were further supported by transwell migration assay. α-H decreased the percentages of invading cells to 62.56%, 41.29%, and 38.49% when cells were treated with 1, 10, and 25 µM of α-H, respectively (**Figure 4B**).

### α-H Inhibited the Activity and Expression of MMP-2 and MMP-9 and Increased TIMP-1 Levels

**Figure 5:**
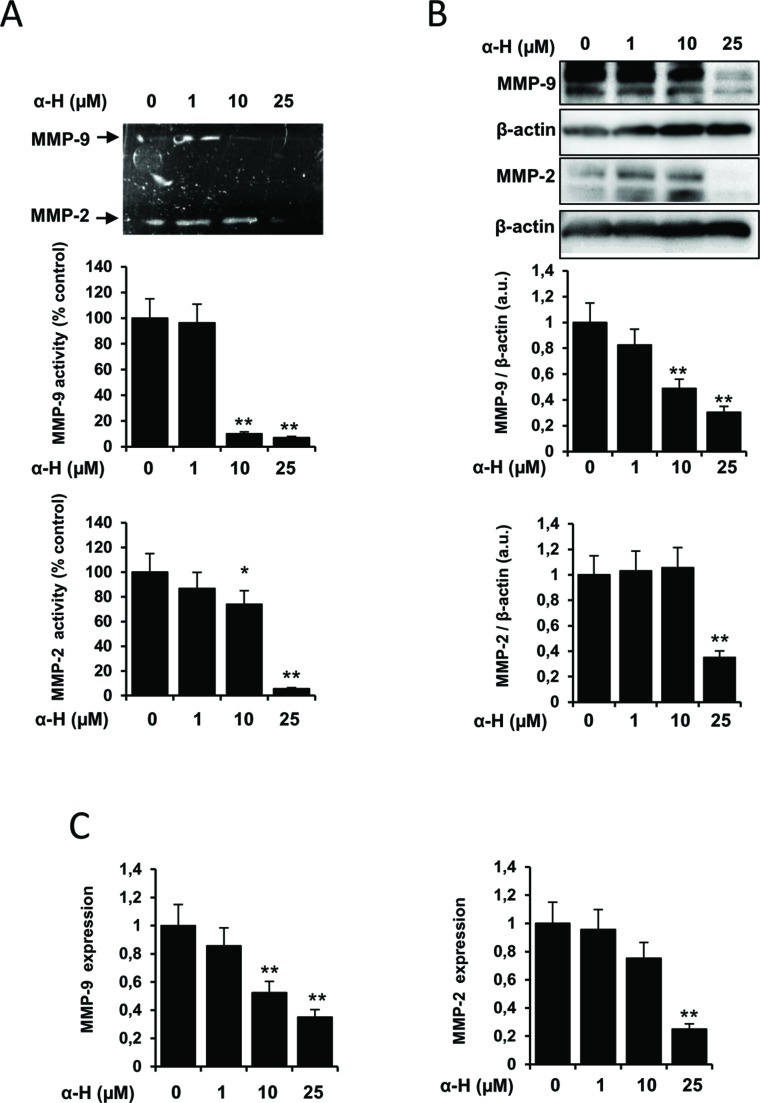
α-H inhibited the expression and activity of MMP-2 and MMP-9. U87 cells were treated with different concentrations of α-H (1, 10, and 25 μM) or vehicle as control for 24 h. **(A)** Conditioned medium was collected, and the activities of MMP-2 and MMP-9 were determined by gelatin zymography assay. A representative experiment is shown of three performed. Bars graphs show densitometry quantification of the bands and represent mean percentage of MMPs activities ± S.D. from three independent experiments. *P < 0.05 and **P < 0.01 with respect to the non-treated cells. **(B)** MMP-2 and MMP-9 protein levels were determined by Western blot. β-actin was used as loading control. A representative experiment is shown of three performed. Bars graphs show densitometry quantification of the bands and represent means MMPs/β-actin ratio ± S.D. from three independent experiments. *P < 0.05 and **P < 0.01 with respect to the non-treated cells. **(C)** mRNA expression of MMP-2 and MMP-9 was determined by quantitative PCR. Values represent the means ± S.D. of three independent experiments performed in triplicate. *P < 0.05 and **P < 0.01 with respect to the non-treated cells.

**Figure 6:**
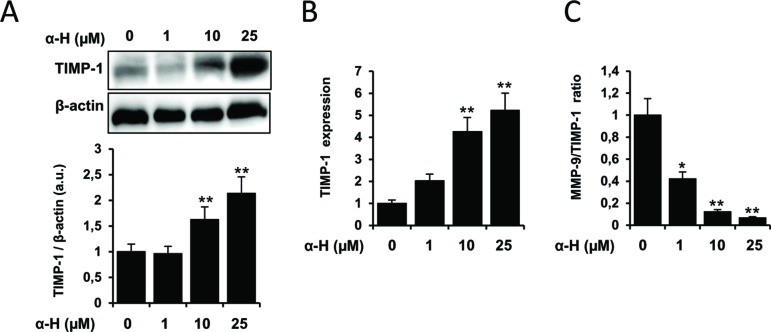
TIMP-1 expression is upregulated after α-H treatment. U87 cells were treated with different concentrations of α-H (1, 10, and 25 μM) or vehicle as control for 24 h. **(A)** TIMP-1 protein levels were determined by Western blot. β-actin was used as loading control. A representative experiment is shown of three performed. Bars graphs show densitometry quantification of the bands and represent means ± S.D. from three independent experiments. **(B)** mRNA expression of TIMP-1 was determined by quantitative PCR. Values represent the means ± S.D. of three independent experiments performed in triplicate. **(C)** MMP-9/TIMP-1 ratio was calculated from data obtained by densitometry. MMP-9 data were from Figure 5B. *P < 0.05 and **P < 0.01 with respect to the non-treated cells.

Matrix metalloproteinases (MMPs) can degrade components of the extracellular matrix facilitating tissue remodeling and cell motility. MMP-2 and MMP-9 have been described to play an important role in the migration, invasion, and metastasis of glioma cells (Nakada et al., 2003). MMP activities are regulated by endogenous tissue inhibitors of metalloproteinases such as TIMP-1, being the MMP/TIMP imbalance important for the tumor cell survival (Bourboulia and Stetler-Stevenson, 2010; Jackson et al., 2016). Therefore, we first investigated whether α-H could regulate the expression and activity of MMP-2 and MMP-9. Enzymatic functions were estimated by gelatin zymography assay. As shown in **Figure 5A**, the activities of MMP-2 and MMP-9 were significantly reduced by α-H treatment. Additionally, α-H also downregulated the protein expression of MMP-2 and MMP-9 (**Figure 5B**) compared with the control condition. In agreement with the downregulation of MMP proteins, mRNA levels of MMP-2 and MMP-9 were also significantly decreased after α-H treatment (**Figure 5C**). By contrast, protein and mRNA expression levels of the inhibitor TIMP-1 increased after exposure of cells to α-H (**Figures 6A, B**). Analysis of the correlation between MMP-9 and TIMP-1 expression showed a significant decrease (**Figure 6C**), suggesting that a reduced MMP-9/TIMP-1 ratio might be associated with the inhibitory effects of α-H.

### p38MAPK Signaling Is Involved in the Inhibition of MMPs by α-H

**Figure 7:**
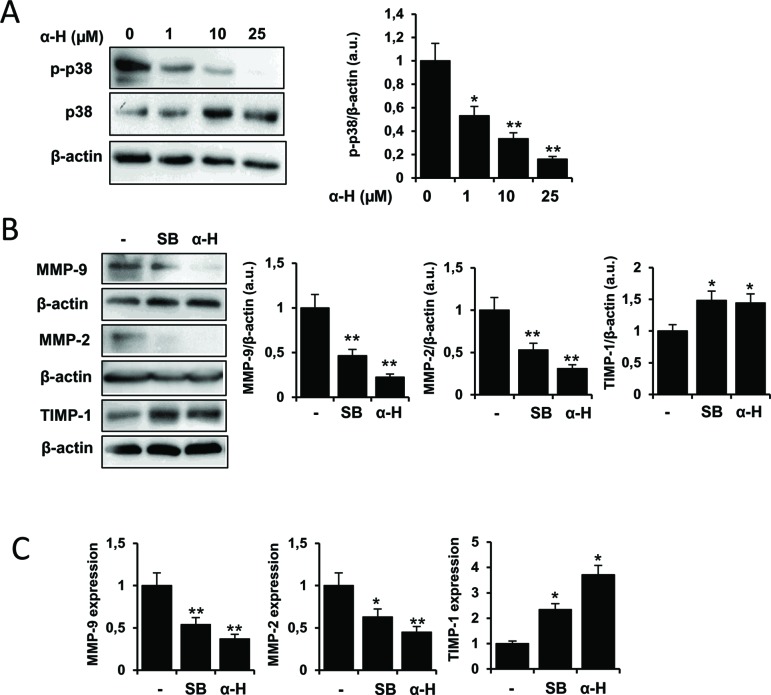
α-H treatment inhibited p38MAPK signal showing similar effects on MMPs and TIMP-1 expression as a specific p38MAPK inhibitor. **(A)** U87 cells were treated with different concentrations of α-H (1, 10, and 25 μM) or vehicle as control for 24 h. p-p38 and p38 protein levels were determined by Western blot. β-actin was used as loading control. A representative experiment is shown of three performed. Bars graphs show densitometry quantification of the bands and represent means ± S.D. from three independent experiments. **(B)** Cells were treated with a specific p38MAPK inhibitor (SB202190, 10 μM) or α-H (25 μM) for 24 h. MMP-9, MMP-2, and TIMP-1 protein levels were determined by Western blot. β-actin was used as loading control. A representative experiment is shown of three performed. Bars graphs show densitometry quantification of the bands and represent means ± S.D. from three independent experiments. **(C)** mRNA expression of MMP-9, MMP-2, and TIMP-1 was determined by quantitative PCR on U87 cells treated as in B. Values represent the means ± S.D. of three independent experiments performed in triplicate. *P < 0.05 and **P < 0.01 with respect to the non-treated cells.

Mitogen-activated protein kinase (MAPK) signaling pathways have been reported to be involved in the regulation of MMPs (Chakraborti et al., 2003). Interestingly, p38MAPK activation has been described to mediate migration of human glioblastoma cells (Park et al., 2002; Gurgis et al., 2015; Sangpairoj et al., 2017), and several natural products exhibited anti-tumoral effects *via* modulation of this pathway (Aroui et al., 2016; Yuan et al., 2019). Therefore, we evaluated whether α-H affected p38MAPK activation. We found that after treatment with α-H, the levels of p-p38 in U87 cells were significantly decreased in a dose-dependent manner (**Figure 7A**). Furthermore, when a specific inhibitor of p38MAPK (SB202190) was used, MMP-2 and MMP-9 protein levels were downregulated, whereas TIMP-1 was increased in a similar manner as after α-H treatment (**Figure 7B**). Additionally, a similar trend was observed in mRNA expression of MMPs and TIMP-1 after p38 inhibition or α-H treatment (**Figure 7C**). These results indicated that α-H probably blocked the activation of MMPs *via* p38MAPK downregulation.

### α-H Exhibited a Significant Inhibition of Tumor Growth and Impaired p38MAPK Signaling and MMP-9 Expression

**Figure 8:**
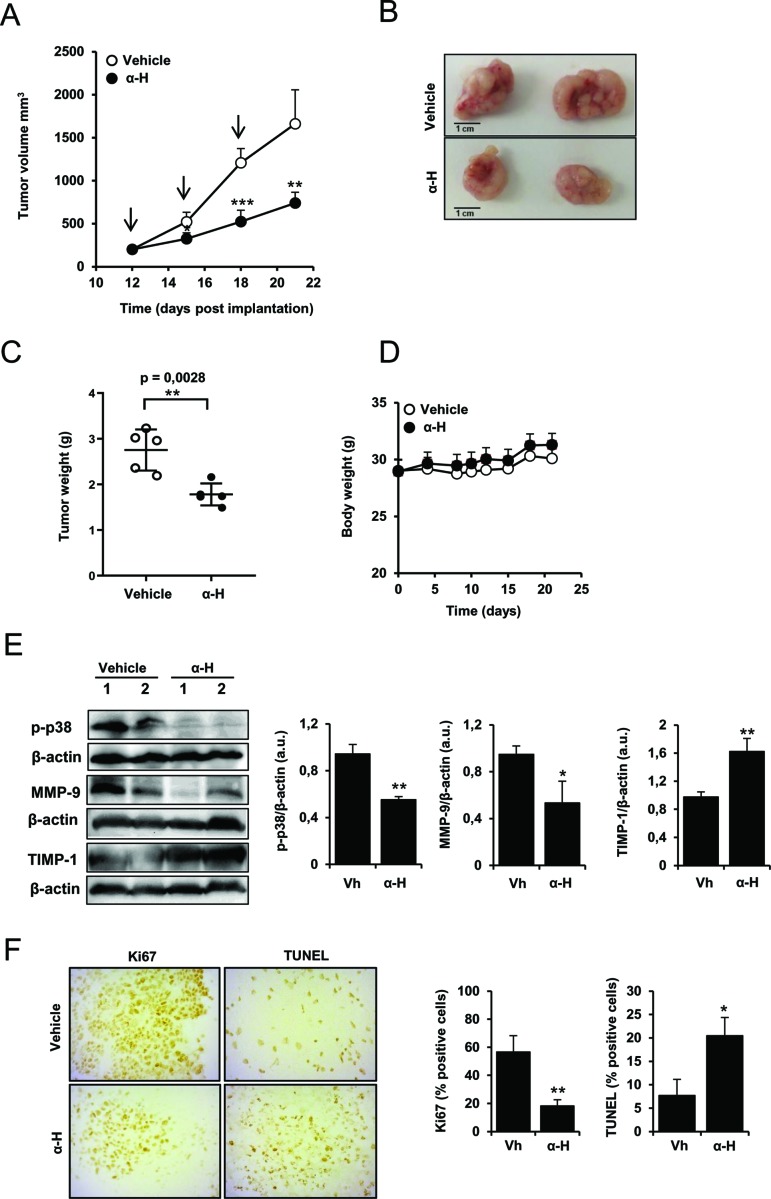
Inhibitory effects of α-H on U87 xenograft growth. U87 cells (3 × 10^6^ cells, 100-µl PBS) were implanted subcutaneously into the right flanks of NSG mice. When tumors reached a volume of ∼200 mm^3^, mice were treated i.v. every 3 days with α-H (1 mg/kg) or with vehicle as indicated by black arrows. After 10 days of treatment, the tumor nodules were isolated and weighted. **(A)** Tumor volume was calculated as described in Material and Methods. Results are the means for groups (n = 5) ± S.D. **(B)** Representative images of tumor nodules harvested on day 21 from each treatment group. Scale bar = 1 cm. **(C)** Weight of tumors isolated on day 21. *P < 0.01 with respect to vehicle group **(D)** Body weight was measured at indicated times. Results are the means for groups (n = 5) ± S.D. **(E)** Expression levels of MMP-9, TIMP-1, and p-p38 were determined by Western blot on tumor tissues of vehicle control and α-H-treated mice. β-actin was used as loading control. Bar graphs show densitometry quantification of the bands and represent means ± S.D of the different animals. **(F)** Immunohistochemical analysis of proliferation marker Ki-67 and TUNEL-positive cells in the tumor tissues (**left panel**). The results shown are representative of randomly selected tumor fields from each specimen. Graphs represent positive cells in IHC staining evaluated as described in Materials and Methods (**right panel**). *P < 0.05, **P < 0.01, and ***P < 0.001 with respect to vehicle group.

In order to evaluate the preclinical effects of α-H, *in vivo* tumor growth was tested using a subcutaneous xenograft model. Tumor volumes were significantly decreased in α-H-treated mice compared with those in vehicle control (**Figures 8A, B**) with a reduction of 55.41% on day 21 (*p* = 0.0048) (**Figure 8A**). At the end of the treatment, the weight of isolated tumors showed a clear reduction on treated group (**Figure 8C**). No toxic effects and no significant change in body weight were observed (**Figure 8D**). Furthermore, consistent with the *in vitro* data, we confirmed that phosphorylation of p38MAPK was suppressed by α-H treatment in tumor tissues and reported the downregulation of MMP-9 and a significant increase in TIMP-1 expression (**Figure 8E**). Moreover, immunohistochemical staining also revealed that α-H treatment inhibited Ki67 proliferation marker compared with control group and induced tumor apoptosis *in vivo* as indicated by the increase in TUNEL positive cells (**Figure 8F**).

Collectively, these *in vivo* results confirm that α-H treatment suppressed GBM tumor growth through similar mechanisms to those reported in the *in vitro* experiments.

## Discussion

Glioblastoma multiforme is the human rare disease most common of primary brain tumor, with a poor clinical outcome due to the uncontrolled proliferation and invasive abilities of the cells (Stupp et al., 2005; Stupp et al., 2009; Onishi et al., 2011). Despite partial advances in therapeutic strategies, the ability of GBM to avoid immunosurveillance and its invasive properties have encouraged the search for the discovery of more effective agents.

Natural products have been currently used for the screening and design of new anticancer therapies, and several plant-derived compounds have been reported to be potential anti-glioma agents (Desai and Bhushan, 2017; Park et al., 2017). Hispanolone is a labdane diterpenoid isolated from *Ballota hispanica*, a plant species widely distributed in Spain (Savona et al., 1978). Several species of the genus *Ballota* are used in folk medicine with therapeutic purposes (Morteza-Semnani and Ghanbarimasir, 2019). Thus, effects as antiemetics, expectorants, diuretics, antispasmodics, antibacterials, sedatives, vermifuges, among others have been described for some of these species. Additionally, various labdane diterpenoids have been reported to possess important biological activities including anti-inflammatory, antiviral, antibacterial, and antitumor properties (Traves et al., 2013; Endringer et al., 2014). Hispanolone derivatives have been shown to exhibit significant cytotoxic and anti-proliferative effects on tumorigenic cell lines (melanoma, leukemia, hepatocellular carcinoma, breast, ovarian, and several types of adenocarcinoma) (Giron et al., 2008; Traves et al., 2013; Mota et al., 2015). In the current study, we found that the hispanolone derivative α-H significantly inhibited cell growth and induced apoptosis in glioblastoma cells. Analysis of cell cycle distribution by flow cytometry revealed that α-H has no relevant effects on cell cycle arrest in U87 cells but caused a remarkable increase in the percentage of cells in sub-G1 phase (apoptotic population) in diterpenoid-treated cells in contrast to the control. Indeed, α-H treatment induced dose-dependent apoptosis as shown, by light field microscopy, typical features of apoptosis as cell shrinkage, chromatin condensation, blebbing, and the formation of apoptotic bodies. Furthermore, flow cytometry analysis using annexin V-FITC demonstrated that treatment with 25-µM α-H increased the percentage of apoptotic cells to 89.01%. Interestingly, α-H treatment did not affect the viability of non-tumoral microglial cells, suggesting selective action on glioblastoma cancer cells.

The ability to induce apoptosis constitutes an important property of an anticancer agent. Apoptosis can be activated by two main pathways, namely, the extrinsic death receptor and the intrinsic mitochondria pathways, where members of the caspase family of cysteine proteases as caspase 8 and caspase 9 play crucial roles, respectively (Elmore, 2007; Logue and Martin, 2008; Ouyang et al., 2012). Both pathways trigger the activation of caspase 3, a key executioner in the apoptotic machinery. Furthermore, both pathways are linked by Bid, a member of the Bcl-2 protein family, which is cleaved by caspase 8 and leads to activation of the mitochondrial pathway. The Bcl-2 family includes both anti-apoptotic (including Bcl-2 and Bcl-xL) and pro-apoptotic (including Bax) members, and it is well known for its key role in the control of apoptosis (Hengartner, 2000). Resistance to apoptosis of tumor cells involves several mechanisms including overexpression of anti-apoptotic proteins (Bcl-2) or downregulation/mutation of pro-apoptotic proteins such as Bax. Indeed, the balance between expression levels of anti-apoptotic Bcl-2 and pro-apoptotic Bax is critical for cell survival and death. In this study, we observed that α-H induced activation of caspase 3, caspase 8, and caspase 9. Moreover, a decrease in the protein levels of Bcl-2 and Bcl-xL was observed, while Bax protein levels were increased following treatment with α-H. As a consequence, the rise in the ratio of Bax to Bcl-2 was significantly enhanced, shifting the balance to the apoptotic status. Interestingly, protein levels of Bid-inactive form were decreased, suggesting that activated caspase 8 cleaved Bid after α-H treatment. Thus, these data provide evidences that the α-H induces apoptosis *via* both the extrinsic and the intrinsic pathways. Our results were consistent with previous studies from our group (Traves et al., 2013) in which hispanolone derivatives (including α-H) have been described to induce apoptosis through activation of death receptor and mitochondria pathway as well as modulation of expression levels of anti-apoptotic proteins (Bcl-2) and pro-apoptotic proteins (Bax) in several carcinoma lines.

Together with resistance to apoptosis, it is well known that angiogenesis and tumor motility are essential for the proliferation and survival of glioma cells (Tate and Aghi, 2009; Onishi et al., 2011). Extracellular matrix (ECM) is considered as an important barrier against glioma metastasis, and its degradation appears to be one of the key events in the complex process of glioblastoma invasion (Forsyth et al., 1999). Matrix metalloproteinases (MMPs) are proteolytic enzymes that play essential roles in the degradation of most components of the ECM such as collagen, laminin, fibronectin, and proteoglycans (Nathoo et al., 2005). In glioma tissues, MMP-2 and MMP-9 are the two MMPs most highly expressed (Forsyth et al., 1999). Indeed, MMP-9 expression has been associated with the degree of malignant glioma, and it is considered as a good predictor of invasive glioma cell growth (Yan et al., 2011; Asuthkar et al., 2012). Therefore, the search for new drugs that control the migratory and invasive capacities of glioblastoma cells is a critical issue. We investigated the activity of MMP-2 and MMP-9 after α-H treatment and found that both MMPs were inhibited as confirmed by gelatin zymography. Further studies demonstrated that α-H also reduced the mRNA and protein expression levels of MMP-2 and MMP-9. In contrast, TIMP-1 expression was upregulated in the presence of α-H. Linked to this, α-H exhibited inhibitory effects on invasion and migration of glioblastoma cells lines, as confirmed in *in vitro* transwell and scratch wound healing assays. In view of our data, it is reasonable to infer that the inhibitory effect of α-H on glioblastoma cell migration and invasion may be associated to MMP-2 and MMP-9 downregulation.

Although several labdane diterpenoids such as sclareol, coronarin D, andrographolide, and selected hispanolone derivatives have been described to exhibit potent antitumor activities *via* induction of apoptosis (Mahaira et al., 2011; Lim et al., 2012; Traves et al., 2013; Mishra et al., 2015; Mota et al., 2015; Wang et al., 2015; Zhang et al., 2017), few data have been reported regarding their role on cell migration. Indeed, to the best of our knowledge, this is the first study describing the anti-invasiveness properties of hispanolone derivatives on glioblastoma tumors. Consistent with our results, the labdane diterpenoid andrographolide has been reported to inhibit the invasion and migration of several tumor cells including breast, colon, lung, and glioblastoma cancer cells through modulation of MMP expression (Shi et al., 2009; Chao et al., 2010; Lee et al., 2010; Chao et al., 2013; Yang et al., 2014; Zhai et al., 2015; Hsieh et al., 2017), whereas coronarin D suppressed TNF-induced cellular invasion *via* downregulation of MMP-9 activity (Kunnumakkara et al., 2008). Interestingly, other natural products including neurostimulants as caffeine or flavonoids such as luteolin or naringin also exhibited inhibitory effects on migration of glioma cells *via* regulation MMP pathway (Cheng et al., 2016; Wang et al., 2017).

Several mechanisms, including MAPKs, JAK/STAT, PI3K/AKT, and NF-κB, have been described to be involved in the modulation of MMP activation and cell migration. In this regard, accumulating evidence have suggested that p38MAPK activation plays a central role in the progression of tumorigenesis and regulation of MMPs and TIMPs in gliomas (Park et al., 2002; Yang et al., 2013). In this study, we found that α-H treatment led to a significant attenuation of p38MAPK phosphorylation. Therefore, through inactivation of p38MAPk signaling, α-H might decrease MMP levels resulting in inhibition of glioma migration. These results were further supported by the effects of specific p38MAPK inhibitor (SB202190). Thus, similar to α-H, SB202190 treatment suppressed the expression of MMPs and upregulated TIMP-1 levels. Indeed, it is not unusual that natural products modulate MMP proteins *via* inactivation of p38MAPK. Compounds as resveratrol, berberine, curcumine, p-cymene, aloe-emodin, or baicalein have been reported to reduce MMP expression through prevention of p38MAPK activation in different cell lines, and others as caffeine and naringin have shown similar effects in glioma cells (Huang et al., 2008; Huang et al., 2011; Cao et al., 2014; Zhang et al., 2014; Yan et al., 2015a; Yan et al., 2015b; Aroui et al., 2016; Li et al., 2016).

Finally, preclinical studies using an *in vivo* xenograft model of glioma showed that administration of α-H effectively inhibited tumor growth without significant off-target effects, establishing a proof of concept for the potential use of α-H as GBM treatment. Furthermore, consisting of the *in vitro* findings, inhibition of MMPs and p38MAPK were also observed in tumor tissues, as well as a reduction in the proliferation index and a significant increase of apoptotic cells.

In summary, our results demonstrate that α-H suppressed proliferation and elicited apoptosis in glioblastoma cells. The underlying mechanisms involved the induction of apoptosis through both extrinsic and intrinsic apoptotic pathways, *via* activation of caspase 3, 8, and 9, and an increased Bax/Bcl-2 ratio. In addition, α-H also increased TIMP-1 expression and inhibited mRNA expression and protein levels of MMP-2 and MMP-9 and hence their enzymatic activities, which may be associated to the reduced migration and invasion capacities of glioblastoma cells exerted by α-H. Mechanistically, attenuation on MMP expression seems to be related with inactivation of p38MAPK pathway. Thus, this is the first report demonstrating that α-H inhibited glioblastoma progression *in vitro* and *in vivo* through implementation of tumor apoptosis and reduction of the metastatic potential of GBM cells. Taking into account these results together with that α-H seems to exhibit a low toxicity in non-tumoral cells and also in preclinical models, our study validate α-H as a promising anti-tumoral drug for GBM therapy.

## Data Availability

All datasets generated for this study are included in the manuscript and/or the **Supplementary files**.

## Author Contributions

V-SM, AL, BH, and SoH designed the research work. V-SM performed the research with LJ-G, PA, SaH, and AL. AA and AE-B performed spectral characterization of the compound. Data analysis, data interpretation, and manuscript preparation were done by AL, BH, and SoH. All authors contributed to manuscript revision and read and approved the submitted version.

## Funding

This study was supported by grant PI11/00036, PI14/00055, and PI17/00012 from the FIS, MPY 1410/09 from ISCIII and Spanish Ministry of Health (Instituto de Salud Carlos III; RD12/0036/0059) to SoH and by grants IERPY 1149/16 and IERPY-M 389/18 to AL. L JG was supported by FIS (FI12/00340). SaH was supported by IERPY 1149/16 from ISCIII.

## Conflict of Interest Statement

BH and SoH are inventors on a Spanish patent application on labdane diterpenoids as anti-tumoral agents. The remaining authors declare that the research was conducted in the absence of any commercial or financial relationships that could be constructed as a potential conflict of interest.
