# Supplementary material for: α-Hispanolol Induces Apoptosis and Suppresses Migration and Invasion of Glioblastoma Cells Likely via Downregulation of MMP-2/9 Expression and p38MAPK Attenuation
Source: Front Pharmacol. 2019 Sep 3;10:935. doi: 10.3389/fphar.2019.00935 (PMC6733979; doi:10.3389/fphar.2019.00935)

Supplementary Material

α-hispanolol induces apoptosis and suppresses migration and invasion of glioblastoma cells likely via downregulation of MMP-2/9 expression and p38MAPK attenuation

Vanesa Sánchez-Martín[1,2], Lidia Jiménez-García[1,3], Sandra Herranz[1], Alfonso Luque[1], Paloma Acebo[1], Ángel Amesty[4], Ana Estévez-Braun[4], Beatriz de las Heras[2,*] and Sonsoles Hortelano[1,*]

*** Correspondence:** shortelano@isciii.es; lasheras@ucm.es

Supplementary Data 1: Procedure for α-hispanolol (α-H) preparation

α-hispanolol (α-H) was obtained from the natural diterpene hispanolone as previously reported (Giron et al., 2008) following the procedure described by Rodríguez-Hahn *et al.* (Rodríguez-Hahn et al., 1995).

Thus 40 mg of hispanolone in 5 mL of EtOH:Dioxane (3:2) were treated with 4 equiv of $NaBH_4$ (19.0 mg) and the reaction mixture was stirred for 4 h, until disappearance of the starting material. Then the reaction mixture was treated with 5 mL of 5% NaOH, and extracted with CH_2Cl_2 (3 x 5 mL). The combined organic layers were washed with brine, dried over $MgSO_4$ and filtered. The solvent was removed under reduced pressure and the crude was purified by preparative-TLC using Hexanes: EtOAc (7:3) to yield 11.4 mg (28%) of α-hispanolol and 2.5 mg (6%) of its epimer β-hispanolol.

α-hispanolol is stable in DMSO since ^{1}HNMR of α-hispanolol in DMSO-d6 run after 24 h and 48 h did not show decomposition.

Supplementary Figure 1: Chemical structure of α-hispanolol (α-H)

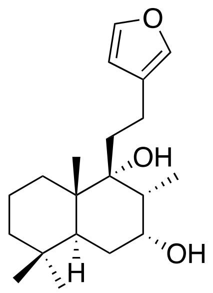

Supplementary Figure 2: ^{1}H NMR ($CDCl_3$, 500 MHz) of α-hispanolol (α-H)

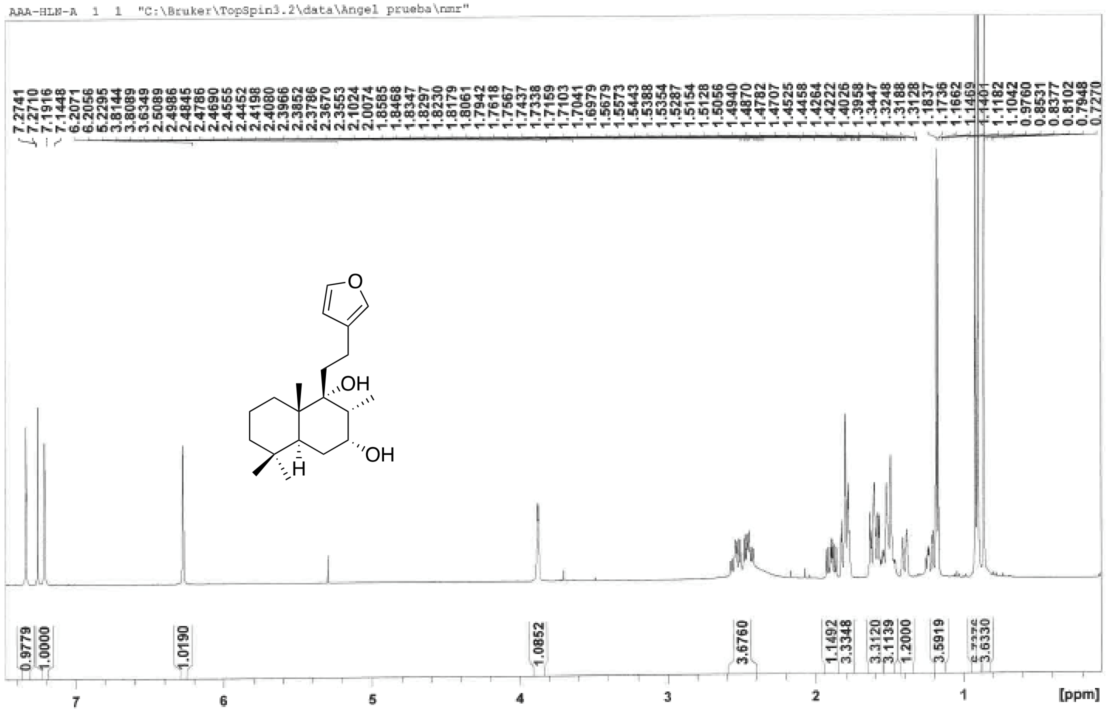

Supplementary Figure 3: ^{13}C NMR ($CDCl_3$, 125 MHz)of α-hispanolol (α-H)

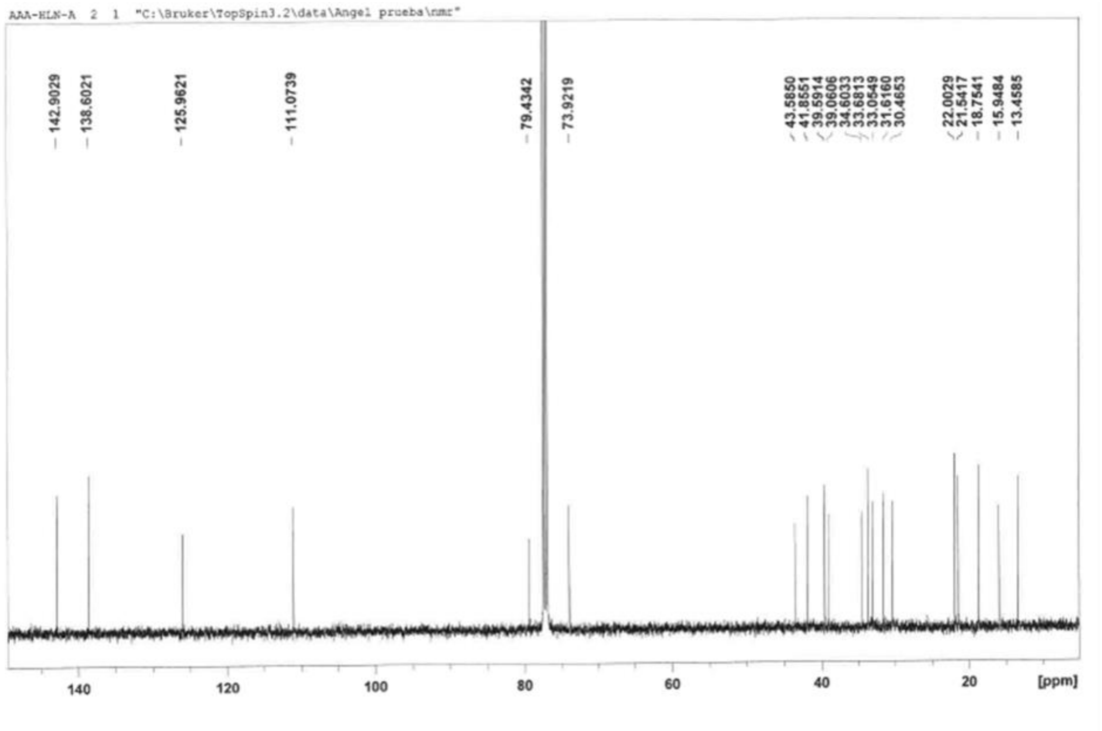

Supplementary Figure 4: Effects of α-hispanolol (α-H) on morphology and apoptosis in U373 cells. (A) Microphotographs were taken after 24 h of incubation with 25 μM α-H or vehicle as control. No signs of characteristics of apoptosis are observed. (Scale bars = 40 μm). Data presented are from one representative experiment out of three. (B) U373 cells were treated with different concentrations of α-H (1-100 μM) or vehicle as control for 24 h. Collected cells were stained with annexin V-FITC and propidium iodide, and then analyzed by flow cytometry. Graph show percentage of apoptotic cells ± S.D. from three independent experiments.

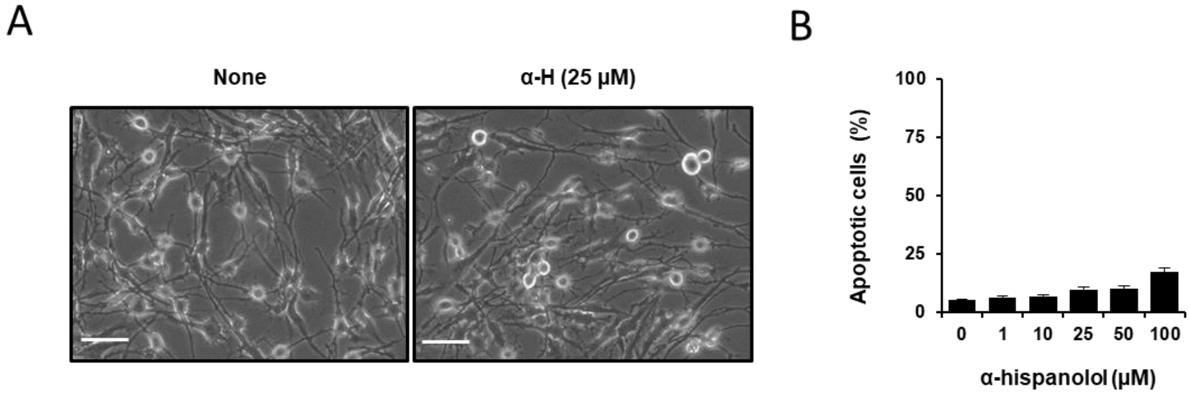

Supplementary Figure 5. α-H did not exhibit significant toxicity in non tumoral microglia cells. BV2 cells were treated with different concentrations of α-H (1-100 μM) for 24h. (A) Cell viability was determined by MTT assay and reported as mean of the cell viability percentage ± S.D. from three independent experiments. (B) Apoptosis was analysed by flow cytometry after stained with annexin V-FITC and propidium iodide. Graph bars show percentage of apoptotic cells ± S.D. from three independent experiments.

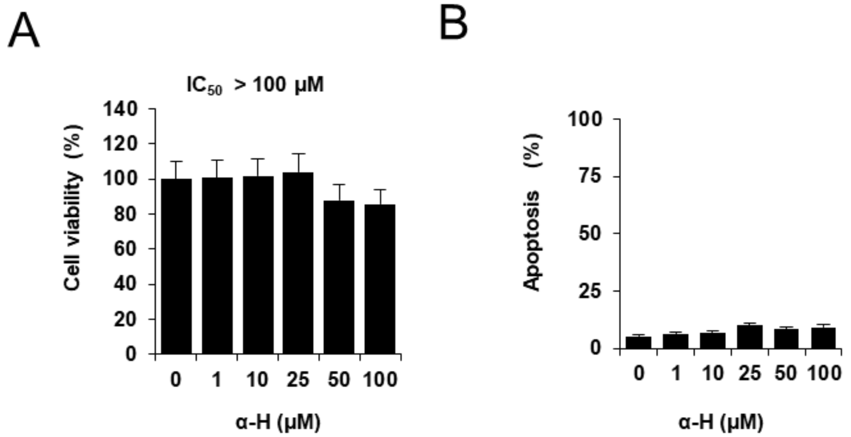

Supplement: Supplementary file 1 [file DataSheet_1.pdf]
